# Data on electroencephalographic activity while exposed to pink noise modified by the frequency responses of three headphone models

**DOI:** 10.1016/j.dib.2021.107528

**Published:** 2021-10-29

**Authors:** Norberto E. Naal-Ruiz, Luz M. Alonso-Valerdi, David I. Ibarra-Zarate

**Affiliations:** Escuela de Ingenieria y Ciencias, Tecnologico de Monterrey, Monterrey, N.L., Mexico

**Keywords:** Neural activity, Frequency response, Pink noise, Acoustic treatments, Inverse filtering

## Abstract

The data described in this article refers to the experimental paradigm where subjects listened to the same pink noise modified by the frequency responses of three headphone models while their brain responses were recorded by means of electroencephalography. Six types of data are described: 1) pink noise sounds with a length of 2.73 s used for the experimental paradigm; 2) electrophysiological recordings, 29 in the first session and 25 in the last session recorded for five minutes; text files with 3) electrode positions and 4) information to identify physiological data and headphone group; 5) a table with technical specifications of the physical headphone model used in the experiment; and 6) a figure summarizing the experimental design. The information in this work can be used to compare the electrical activity of the brain in acoustic treatments where headphones are a key element to meet the therapeutic effect.

## Specifications Table

**Table:** 

Subject	Biological sciences
Specific subject area	Neuroscience: Sensory systems
Type of data	Sounds – pink noise filtered with the frequency responses of three different headphone models. Electrophysiological recordings – signals recorded by means of electroencephalography. Text files – electrode positions, and information to identify physiological data and headphone group. Table – technical specifications of Atvio® headphones (ATVIO). Figure – experimental design.
How data were acquired	*Sounds* Pink noise files created and filtered in MATLAB and down sampled in REAPER. Focusrite® Scarlett 2i4 audio interface. Shure® mx150 microphone calibrated to the response of a dbx® RTA-M microphone. Sounds were processed with the characterized frequency responses and inverse filters of the following headphones: • ATVIO • Shure®SRH1840 (SHURE) • Apple® Earpods® (APPLE) *Electrophysiological recordings* 24-channel EASYCAP electrode cap following the 10/20 international system. SMARTING EEG Bluetooth amplifier. OpenViBE software for signals acquisition.
Data format	Sounds – filtered. Electrophysiological recordings – raw. Electrode positions and participants information – text.
Parameters for data collection	The frequency responses of headphone models used to create the inverse filters were measured in an isolation booth at the digital music production studios of Tecnologico de Monterrey, Monterrey Campus. Electrophysiological recordings from participants were collected if they fulfilled the following criteria: ages 19 – 24, currently studying at university level or one-year graduate from university level, no hearing or neurological disorders, and no musical lectures, ear training, or playing an instrument for more than five years.
Description of data collection	The frequency responses were collected at a sample rate of 96 kHz, and the final audio files were down sampled to 44.1 kHz. Electrophysiological recordings of participants with eyes closed were acquired before (first session) and after 30 days of daily exposure to pink noise (last session) in four modalities: (1) in a resting state (baseline), (2) listening to pink noise in ATVIO, (3) listening to pink noise in SHURE, and listening to pink noise in APPLE.
Data source location	Institution: Tecnologico de Monterrey City/Town/Region: Monterrey State: Nuevo Leon Country: Mexico Latitude and longitude: 25.647903503278236, −100.28928530251193
Data accessibility	Sounds, electrophysiological recordings, and text files are available in a public repository. Repository name: Mendeley Data Direct URL to data: https://data.mendeley.com/datasets/9dmd8sr3ht/1 Table: With the article Figure: With the article
Related research article	N.E. Naal-Ruiz, L.M. Alonso-Valerdi, D.I. Ibarra-Zarate, Frequency responses of headphones modulate alpha brain oscillations related to auditory processing, Appl. Acoust. 185 (2022) 108415. https://doi.org/10.1016/j.apacoust.2021.108415.

## Value of the Data

• The dataset contains the effect of frequency responses of headphones in the brain activity of subjects while listening to pink noise, a type of noise adjusted to the human hearing, and which frequency spectrum is observed in natural processes.
• Researchers studying acoustic treatments can benefit from these data because the effect of frequency responses of audio devices in neural activity can be observed.
• Data can be used to compare audio devices or sounds with different frequency content to improve acoustic therapies.

## 1 Data Description

This section describes how data is arranged in the Mendeley Data repository. Additionally, technical information of ATVIO is given in Table 1. Specifications of SHURE and APPLE are available in [3,4].

**Table 1:** Technical specifications of ATVIO. The information is described in the headphones packaging.

**Model**	Atvio®
**Type**	Supra-aural headphones
**Power Capacity**	3 mW
**Impedance**	32 Ω
**Frequency range**	20 Hz – 20 kHz
**Cable length**	1.2 m
**Additional information**	Integrated microphone

### 1.1 Sounds

Pink noise is a colour of noise with reduced magnitude in the frequency spectrum by 3 dB each octave, giving the listener the impression of an equally loud, or flat, sound [1]. The human ear integrates logarithmically, and therefore, this type of noise balances the perceived loudness of the listener. Furthermore, natural processes have been shown to have similar content in the frequency domain [2,3]. This folder contains:

○ PINK.wav – 2.73 s pink noise file in waveform format (WAV) generated with the MATLAB code from [4].
○ ATVIO – subfolder containing 30 pink noise audio files with a length of 2.73 s. These sounds emulate the frequency response of the 30 ATVIO models used. Sounds are provided in this form to show researchers the natural sound of the headphones. However, the sounds listened through these headphones in the experiment were not filtered.
○ SHURE – subfolder containing 30 pink noise audio files in WAV with a length of 2.73 s emulating the sound of SHURE.
○ APPLE – subfolder containing 30 pink noise audio files in WAV with a length of 2.73 s emulating the sound of APPLE.

Each subfolder contains:

○ ID_HP.wav – audio files for each subject with identification number (ID) calibrated to headphone HP (ATVIO, SHURE or APPLE).

Audio files are meant to be heard in ATVIO. Therefore, they might sound unbalanced in other headphone models. It is recommended to listen to the sounds on a digital audio workstation.

### 1.2 Electrophysiological recordings

Data corresponds to the spontaneous activity of 30 participants in four modalities: 1) in a resting state; 2) listening to pink noise in ATVIO; 3) listening to pink noise in emulated SHURE; and 4) listening to pink noise in emulated APPLE. Spontaneous activity is observed when subjects do not perform a specific task while exposed to a constant stimulus, or during a resting state [5]. Neural activity is recorded for extended periods (i.e. 5 minutes) to reduce artifacts during signal acquisition. These signals are later analyzed in the time-frequency domain. It is common to record a resting state, usually referred to as baseline, to later reference activity during constant stimulation, and observe neural synchronization and desynchronization. The signals in all conditions were acquired with eyes closed. Twenty-nine general data format (GDF) files are given for the first session and 25 for the last session. That is because not all the participants continued in the experimental procedure, or session recordings were not useful due to many artifacts. Only the GDF file of participant ID 10 in the last session was included because the first session file had several recording errors.

*First session*. This folder contains subfolders with the electrical activity of the brain recorded in the first session in four modalities:

○ BASELINE – folder with 29 GDF files of subjects in a resting state.
○ ATVIO – folder with 29 GDF files of subjects listening to pink noise in ATVIO.
○ SHURE – folder with 29 GDF files of subjects listening to pink noise in emulated SHURE.
○ APPLE – folder with 29 GDF files of subjects listening to pink noise in emulated APPLE.
  ■ Each folder contains:
    • ID.gdf – GDF file with the electrical activity of the brain recorded in 24 electrodes for five minutes of subject ID.

*Last session.* This folder has 25 GDF files of the electrical activity of the brain recorded in the last session in four modalities. Information is arranged in the same form as in the first session.

### 1.3 Text files

*Participants*. The file *participants.txt* provides information on the ID of GDF and audio files of the first and last sessions for every participant. It shows the assigned group of headphones as well where participants were placed, including their sex, and the heart rate measured before the recording. Columns with an “X” show the available data per session and participant.

*Channels.* The file *channels.txt* provides the name of the electrodes and the position in theta/phi-coordinates (second and third column, respectively).

## 2 Design, Materials and Methods

### 2.1 Calibration of sounds

Frequency responses of headphones were measured in MATLAB with maximum length sequence method playing white noise through each headphone. Emitted sounds were recorded with a calibrated Shure mx150 microphone connected to a Focusrite Scarlett 2i4 audio interface. The sample rate was set to 96 kHz. Then, the frequency response was corrected as if measured with pink noise.

The methodology for the characterization of headphones is described in [6]. This implementation is based on the works of [7,8]. An inverse filter of the frequency response of headphones is created to calibrate the response curve from one model (ATVIO) to other (SHURE or APPLE) by modifying the frequency content of pink noise. ATVIO, SHURE, and APPLE were measured in an isolation booth. The sample rate of all audio files in the repository is 44.1 kHz. This approach reduced the cost of experimentation by approximating the output signal of ATVIO headphones to either SHURE or APPLE. Finally, audio files for the experimental design were down sampled in REAPER digital audio workstation.

### 2.2 Electrophysiological recordings

A 24-channel EASYCAP electrode cap in line with the 10/20 international system and a SMARTING EEG Bluetooth amplifier were used. Data was recorded using OpenViBE software. The sample rate of GDF files is 250 Hz.

### 2.3 Experimental design

**Fig. 1:**
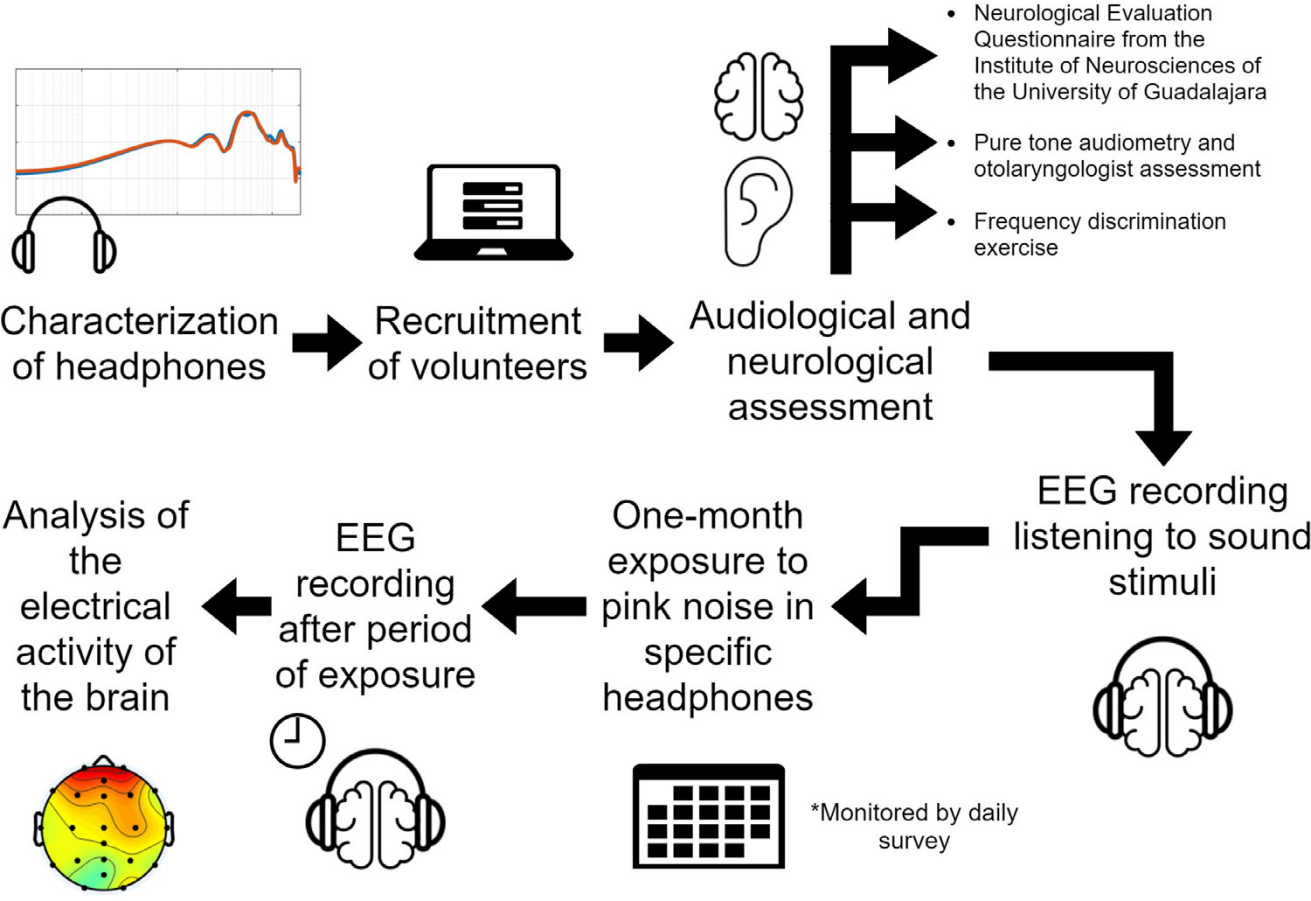
Experimental design. The experiment proceeded in seven steps: from the characterization of headphones to analysis of the electrical activity of the brain. EEG: electroencephalography.

Spontaneous activity of 29 subjects for the first session and 25 for the last session was included in the dataset. Data of participants that fulfilled the following criteria were selected: ages 19–24, currently studying at university level or one-year graduate, no hearing or neurological disorders, and no musical lectures, ear training, or playing an instrument for more than five years. Volunteers were recruited through an online registration form. Then, inclusion criteria were applied based on the results of the Neurological Evaluation Questionnaire from the Institute of Neurosciences of the University of Guadalajara, a pure-tone audiometry, a hearing assessment by an otolaryngologist, and a frequency discrimination exercise. Only data ID 06 is from a subject presenting slight hypoacusis at 250 Hz. However, it was not excluded in accordance with the World Health Organization grades of hearing impairment. Fig. 1 summarizes the experimental design.

First, the researcher asked the participant to remove objects that could interfere with the acquisition equipment. Following, the researcher cleaned the front of the head, the vertex, and areas behind the earlobes of the subject. Afterward, the researcher placed the electrode cap on the head of the participant, adjusted it, connected the Bluetooth device to the cap, and injected hydrogel to reduce the impedance on the scalp. Signals were routed to the acquisition server of the OpenViBE application. Finally, data acquisition began by recording the electrical neural activity of participants in a resting state for five minutes with eyes closed.

The electrical neural activity of participants was recorded for five minutes in three conditions, listening to pink noise through the three headphone models with eyes closed: ATVIO, SHURE and APPLE. The sounds with a length of 2.73 s were looped for five minutes. Electrophysiological activity and sound were respectively recorded and played simultaneously. At the end of the recording session, each subject was given a pair of ATVIO and 30 files with a 20-minute version of the sounds to be heard daily for a month. Participants were divided into three groups according to the headphone models. Thus, each subject heard the pink noise files through the frequency response of one of the following models: ATVIO, SHURE, or APPLE. The assigned groups are marked in *participants* text file. Subjects had to fill in a daily questionnaire to prove that they listened to the sound on the assigned day. After the implementation period, a final recording session was conducted following the same procedure as the previous session.

## Ethics Statement

The experimental design was approved by the Ethics Committee of Tecnologico de Monterrey. Participants filled in an informed consent before experimentation.

## CRediT Author Statement

**Norberto E. Naal-Ruiz:** Data acquisition, Methodology, Writing - Original Draft. **Luz M. Alonso-Valerdi.**: Conceptualization, Writing - Reviewing and Editing, Validation. **David I. Ibarra-Zarate:** Formal analysis, Validation, Writing - Reviewing, and Editing.

## Declaration of Competing Interest

The authors declare that they have no known competing financial interests or personal relationships which have or could be perceived to have influenced the work reported in this article.
